# Clonal population expansion of *Staphylococcus aureus* occurs due to escape from a finite number of intraphagocyte niches

**DOI:** 10.1038/s41598-023-27928-2

**Published:** 2023-01-21

**Authors:** Grace R. Pidwill, Josie F. Pyrah, Joshua A. F. Sutton, Alex Best, Stephen A. Renshaw, Simon J. Foster

**Affiliations:** 1 School of Biosciences, University of Sheffield, Sheffield, S10 2TN UK; 2 Florey Institute, University of Sheffield, Sheffield, S10 2TN UK; 3 The Bateson Centre, University of Sheffield, Sheffield, S10 2TN UK; 4 School of Mathematics & Statistics, University of Sheffield, Sheffield, S3 7RH UK; 5 Department of Infection, Immunity and Cardiovascular Disease, Medical School, University of Sheffield, Sheffield, S10 2RX UK

**Keywords:** Microbiology, Bacteriology, Cellular microbiology, Pathogens

## Abstract

*Staphylococcus aureus* is a human commensal and also an opportunist pathogen causing life threatening infections. During *S. aureus* disease, the abscesses that characterise infection can be clonal, whereby a large bacterial population is founded by a single or few organisms. Our previous work has shown that macrophages are responsible for restricting bacterial growth such that a population bottleneck occurs and clonality can emerge. A subset of phagocytes fail to control *S. aureus* resulting in bacterial division, escape and founding of microabscesses that can seed other host niches. Here we investigate the basis for clonal microabscess formation, using in vitro and in silico models of *S. aureus* macrophage infection. Macrophages that fail to control *S. aureus* are characterised by formation of intracellular bacterial masses, followed by cell lysis. High-resolution microscopy reveals that most macrophages had internalised only a single *S. aureus,* providing a conceptual framework for clonal microabscess generation, which was supported by a stochastic individual-based, mathematical model. Once a threshold of masses was reached, increasing the number of infecting bacteria did not result in greater mass numbers, despite enhanced phagocytosis. This suggests a finite number of permissive, phagocyte niches determined by macrophage associated factors. Increased understanding of the parameters of infection dynamics provides avenues for development of rational control measures.

## Introduction

Although *S. aureus* is a natural part of the human microflora, particularly on the skin and in the nose, it is also a human pathogen causing a range of diseases from skin and soft-tissue infections to potentially deadly bacteraemia^1–3^. The rise of antibiotic-resistant *S. aureus* strains causes an additional burden to healthcare settings, making effective treatment of *S. aureus* infections complex^4^.

*S. aureus* often enters the body through a breach in host physical defences, such as a wound, where it encounters macrophages and neutrophils, a first line of defence of the host immune system. Mammalian models of systemic disease have demonstrated that if *S. aureus* gains access to the blood, it is transported to the liver, where initial infection is controlled by resident macrophages, called Kupffer cells^5^. We have previously described a “population bottleneck”, whereby phagocytes control all but a small fraction of the *S. aureus* population, resulting in limited bacterial release, whereby those surviving bacteria are able to divide, clonally expand and then spread to other host sites^6–8^. A similar population bottleneck has been observed for other bacterial species^9,10^. Importantly, the population bottleneck phenomenon is observed in multiple *S. aureus* infection models including zebrafish and murine bloodstream infections^6–8^.

During murine bloodstream infection, Kupffer cells have been identified as the cellular effector of the *S. aureus* population bottleneck^6,11^, where depletion of these phagocytes in vivo leads to loss of the bottleneck, alongside enhanced susceptibility to infection^6^. However, systemic spread to distant organs was still observed^6^. Depletion of neutrophils in vivo reduced dissemination to other organs from the liver, although the population bottleneck remained^6^, highlighting the role of neutrophils in the systemic spread of *S. aureus*^12^.

Being able to predict the outcome of disease and the effect of interventions requires the establishment of robust models of disease. Mathematical models have increasingly been used to investigate host–pathogen interactions^13,14^. In previous mathematical models macrophages have been demonstrated to be important for limiting *S. aureus* replication, where a high inoculum was able to overwhelm macrophages^15,16^. However, in vivo*,* macrophages which failed to successfully kill intracellular bacteria underwent lysis, releasing *S. aureus*, which was then phagocytosed by nearby macrophages^16^.

A key facet of the macrophage arsenal employed to combat *S. aureus* are reactive oxygen species (ROS). ROS are pivotal in a phenomenon termed “augmentation” where the presence of commensal organisms, or their components, within the *S. aureus* inoculum ameliorates ROS within macrophages leading to increased bacterial survival^17^. This is important as *S. aureus* will generally infect the host by emerging from the current microflora of the host environment.

Research into the immune-mediated bacterial population bottleneck and clonal expansion during *S. aureus* infection has largely been conducted using in vivo models^6,7^. This has firmly established the phenomenon as a key, early aspect of disease and that macrophages are involved. However, to dissect the relationship between *S. aureus* and macrophages, here we have utilised an in vitro macrophage model of *S. aureus* interaction, using an experimental and modelling approach^17^. We show that clonality is host-dependent, as increasing the multiplicity of infection (MOI) did not enhance numbers of bacterial masses formed. We also show that clonality arises originally at the point of phagocytosis, where most macrophages take up only individual bacteria. The internalised bacteria are mostly controlled but can rarely divide and form masses within macrophages that then emerge to initiate clonal microabscess formation, with potential catastrophic consequences for the host.

## Results

### *S. aureus* clonal masses generally originate from a single bacterium during macrophage infection

Previous work has described a model for *S. aureus*-macrophage interactions^6^. Upon infection of a host, *S. aureus* are phagocytosed by macrophages, with the vast majority of bacteria being effectively killed. A common scenario would be that *S. aureus* enter the body through a breach via a wound in the skin and be carried in the blood to the liver where they are phagocytosed by Kupffer cells. However, under some circumstances, a small proportion can survive macrophage killing mechanisms. This population bottleneck allows clonal expansion of the surviving bacteria, dissemination to distant sites of the body and continued infection.

**Figure 1:**
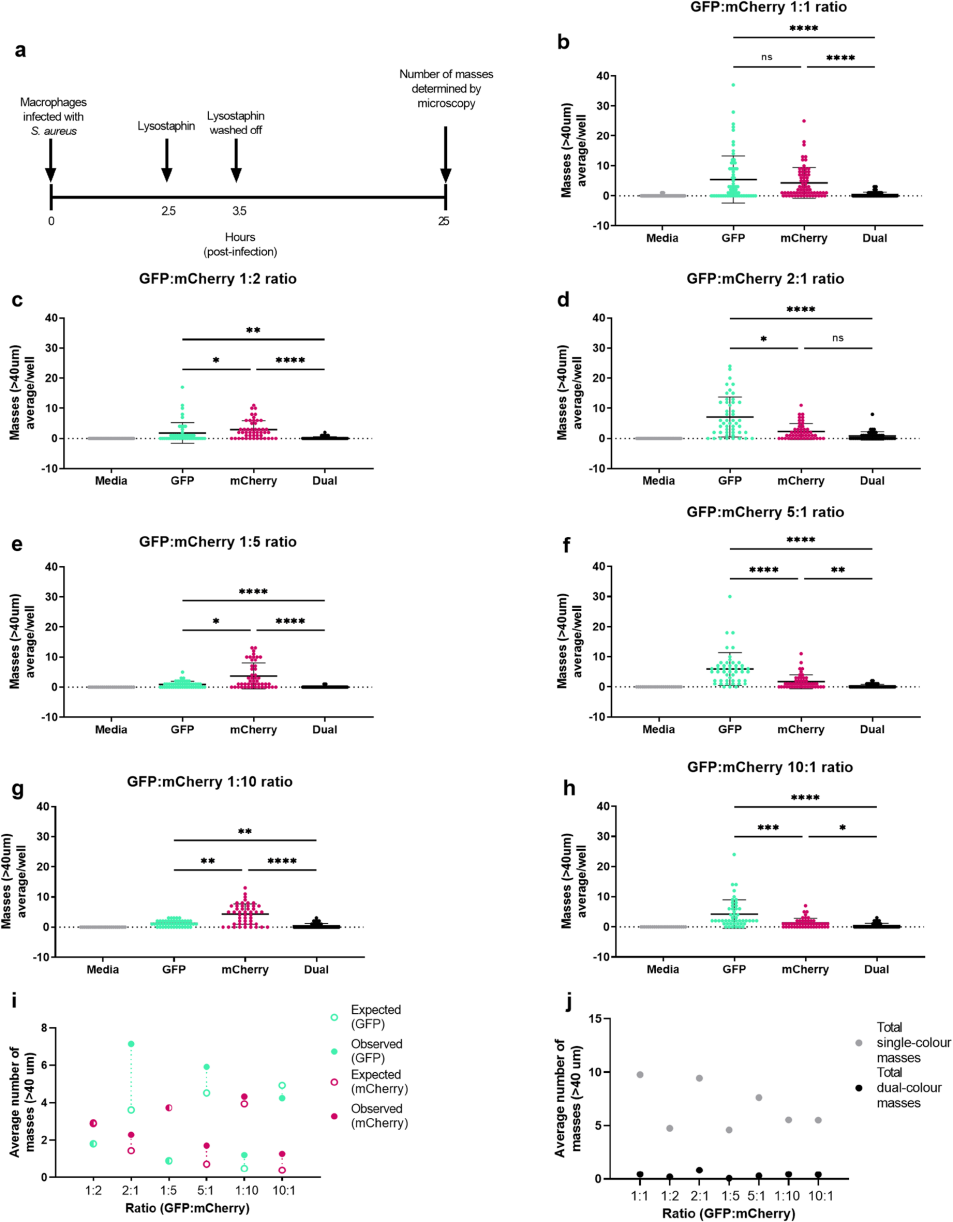
Altering the ratio of GFP:mCherry *S. aureus* influences resulting bacterial mass formation. (**a**) Diagram showing experimental overview. (**b**–**h**) RAW264.7 macrophages were infected with different ratios of GFP and mCherry marked *S. aureus* (total MOI 5). The number of single- or dual-colour bacterial masses detected at 25 h post-infection is indicated, error bars show mean values ± standard deviation, each data point represents a well. Ratios (GFP:mCherry): (**b**), 1:1 ratio (*n* = 4); (**c**) 1:2 ratio (*n* = 4); (**d**) 2:1 ratio (*n* = 4); (**e**) 1:5 ratio (*n* = 3); (**f**) 5:1 (*n* = 3); (**g**) 1:10 (*n* = 3); (**h**) 10:1 (*n* = 3), *p < 0.05, **p < 0.005, ***p < 0.0005, ****p < 0.0001, as determined using a Kruskal–Wallis test with Dunn’s post-hoc test. GFP (cyan), mCherry (magenta), media control (grey), dual (black). **i**, Comparison between expected (open circles) and observed (closed circles) mass numbers for ratio data presented in (**c**–**h**), with expected plots calculated based on 1:1 ratio data from **b**, dotted line depicts difference between expected and observed values. (**j**–**k**), Comparison between number of total single-colour (grey) and dual-colour masses (black) for ratio data presented in (**b**–**h**): (**j**), summary of average plots for (**b**–**h**).

We have previously established a macrophage infection assay, using both cultured RAW264.7 cells and human monocyte derived macrophages (MDMs), which leads to the formation of bacterial masses^17^, as depicted in Fig. 1a. To demonstrate that masses are initially derived from within individual macrophages we have previously used time course studies^17^. Here using time lapse microscopy over a period of 14 h, we show intracellular *S. aureus* NewHG-GFP mass formation within an MDM resulting in macrophage lysis (Movie S1). To test whether these masses are formed via clonal expansion (i.e., founded by a single bacterium) we infected macrophages with two fluorescently marked strains to examine whether masses containing both fluorescent markers were present. The two strains were from the same isogenic background strain, NewHG, with an antibiotic resistance marker (erythromycin (Ery) or kanamycin (Kan)) allowing their quantification by plating on appropriate selective agar^6^. In addition, each strain contained a plasmid encoding a fluorescent reporter along with a tetracycline (Tet) resistance marker. Specifically, the strains were NewHG-GFP(Kan/Tet) and NewHG-mCherry(Ery/Tet). The GFP and mCherry fluorescent strains used were confirmed to have comparable growth rates, cultured alone (Figure S1a), and in competitive culture (Figure S1b). Furthermore, as the fluorescence was plasmid based, we confirmed that each bacterial strain retained the plasmid in antibiotic free culture for the duration of experiments (Figure S1c,d,e), demonstrating that our assay would be able to detect all bacterial masses formed.

Infection of macrophages with *S. aureus* GFP and mCherry strains in equal proportions (1:1 ratio, Fig. 1b) led to similar numbers of single-colour green or red bacterial masses forming, with significantly fewer dual-colour masses. To probe the reason for this skewing of strains away from the injected proportions, we altered the ratio of the two marked strains added to macrophages to 1:2 (Fig. 1c,d), 1:5 (Fig. 1e,f) and 1:10 (Fig. 1g,h). The number of masses observed (1c,d,e,f,g,h) was compared to the numbers predicted (by simple calculation) for 1:2, 1:5 and 1:10 strain ratios (Fig. 1i) based on 1:1 ratio data of GFP:mCherry bacteria (Fig. 1b). For all ratios tested, the fluorescent strain in the higher proportion formed significantly more single-colour masses, with numbers similar to that predicted for the ratios tested (Fig. 1i). The numbers of dual-colour masses consistently remained low irrespective of the ratios of GFP and mCherry bacteria (Fig. 1j).

### Macrophages phagocytose few *S. aureus* by 2.5 h post-infection

**Figure 2:**
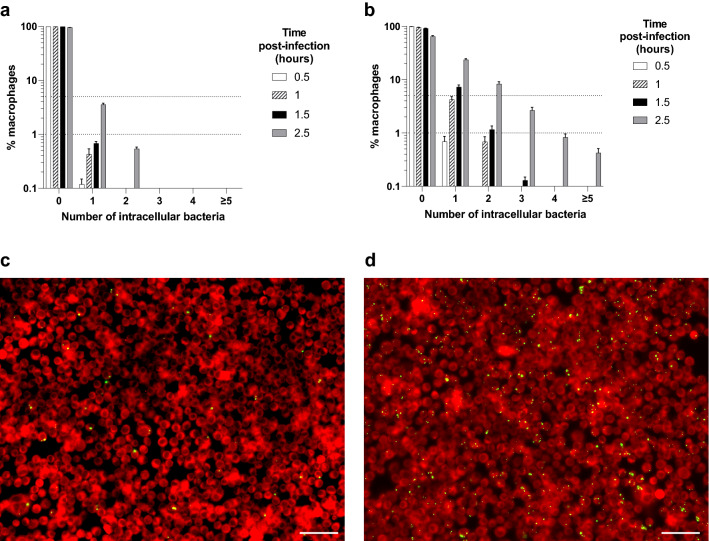
Phagocytosis of *S. aureus* by macrophages over time. (**a**–**b**) Percentage (%) of RAW264.7 macrophages with different numbers of internalised bacteria after incubation with *S. aureus* for 0.5 h (white bars), 1 h (shaded bars), 1.5 h (black bars) and 2.5 h (grey bars). Bars represent mean percentage ± SEM (*n* = 3), dotted horizontal lines show 1% and 5%. (**a**) *S. aureus* MOI 5; (**b**) *S. aureus* MOI 50. (**c**–**d**), representative images of RAW264.7 macrophages (red) with internalised bacteria after incubation with GFP *S. aureus* (green) for 2.5 h: (**c**) MOI 5 *S. aureus*, (**d**) MOI 50 *S. aureus* (scale bars 50 µm).

We next examined at what point the bacterial population bottleneck occurs. There are several possible scenarios including: at the point of phagocytosis, or within a single phagocyte in which most bacteria are killed, or multiple bacteria are phagocytosed but as a rare event a single bacterial cell is treated differently. To address this important question, we quantified how many bacteria are phagocytosed per macrophage at different multiplicities of infection (MOI). In a modification of the mass formation experiments, bacteria were incubated with macrophages for short periods before extracellular bacteria were killed using lysostaphin. After fixation and staining, the number of phagocytosed bacteria was assessed microscopically (Fig. 2). Over time, the number of bacteria phagocytosed increased, however, even at 2.5 h post-infection most macrophages had phagocytosed zero bacteria, with only 3.6% of MOI 5-infected macrophages containing one *S. aureus*, and 0.5% containing two (Fig. 2a). Increasing the *S. aureus* MOI to 50 raised the percentage of macrophages with phagocytosed bacteria, with an average of 23.5% containing one *S. aureus* and 8.3% containing two by 2.5 h post-infection (Fig. 2b). Importantly, as the mass formation assay (detailed in Fig. 1a) involves addition of lysostaphin at 2.5 h post-infection, only bacteria which have been phagocytosed by this time were able to divide to form bacterial masses shown in Fig. 1.

### Centrifugation of bacteria onto macrophages increases phagocytosis of *S. aureus*

**Figure 3:**
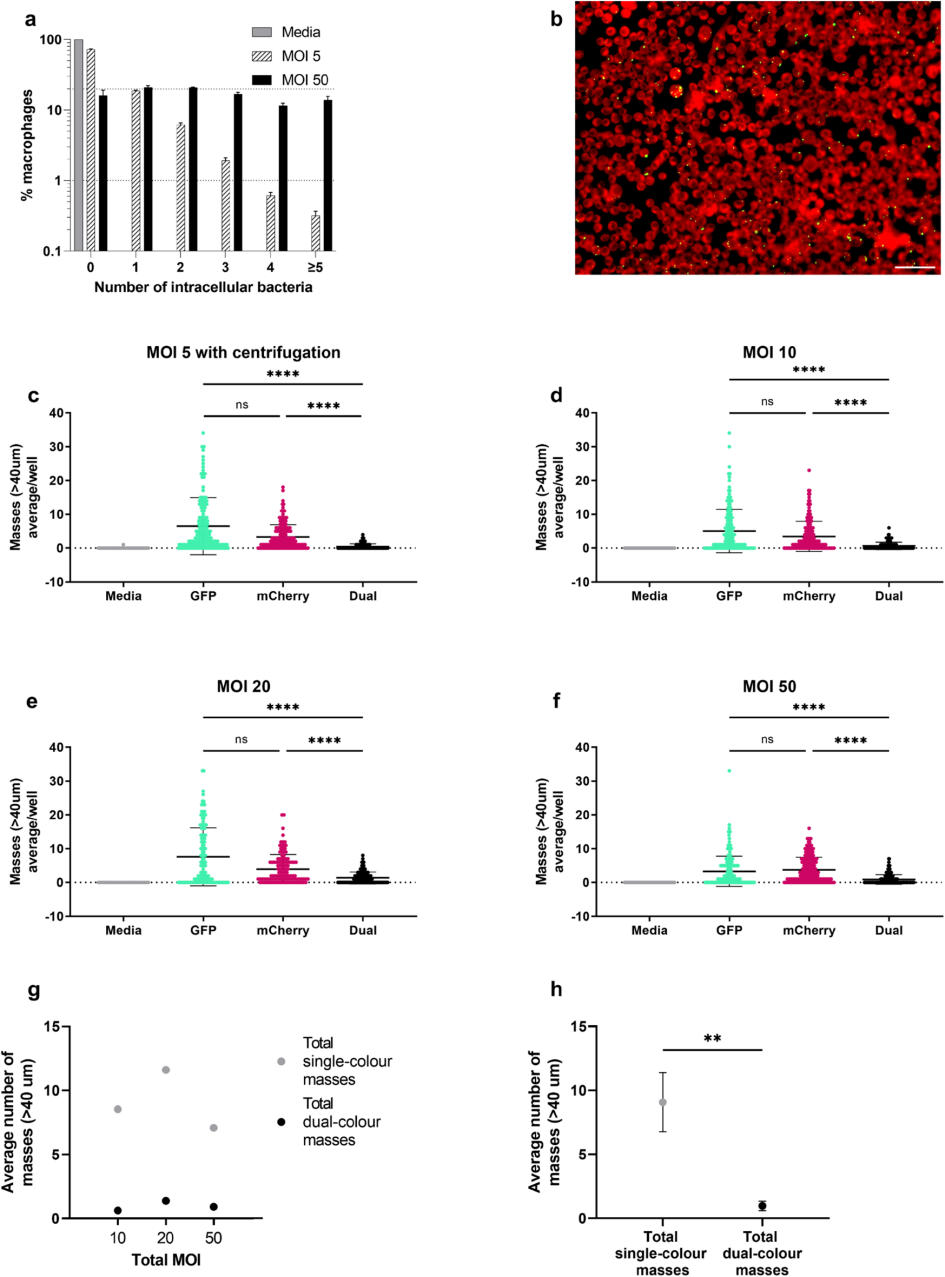
Numbers of bacterial masses does not scale with MOI. (**a**) Percentage (%) of RAW264.7 macrophages with internalised bacteria. Media (grey bars), *S. aureus* MOI 5 (shaded bars) or MOI 50 (black bars) was added to macrophages, centrifuged onto cells and incubated until 2.5 h post-infection, bars represent mean percentage ± SEM, with dotted lines showing 1% and 20% (*n* = 4). (**b**) representative image of RAW264.7 macrophages (red) with internalised bacteria after GFP *S. aureus* (green) were centrifuged onto cells and incubated for 2.5 h at MOI 5, (scale bar 50 µm). (**c**–**f**), RAW264.7 macrophages were infected with GFP and mCherry *S. aureus* in a 1:1 ratio with a total MOI as designated. The number of single- or dual-colour bacterial masses detected at 25 h post-infection is indicated, showing mean values ± standard deviation; (**c**) *S. aureus* (MOI = 5) was centrifuged onto cells before incubation (*n* = 4); (**d**) MOI = 10 (*n* = 4); (**e**) MOI = 20 (*n* = 3); (**f**) MOI = 50 (*n* = 5), GFP (cyan), mCherry (magenta), media control (grey), dual (black) Cells in d-f were uncentrifuged. (**g**–**h**), Comparison between number of total single-colour (grey) and dual-colour masses (black) for ratio data presented in (**d**–**f**); (**g**) summary of average plots for (**d**–**f**); (**h**) total single-colour and dual-colour masses were combined from (**d**–**f**), data are presented as mean ± standard deviation, **p < 0.005, ****p < 0.0001, as determined by Kruskal–Wallis test with Dunn’s post-hoc test for panels (**d**–**g**) or two-tailed Mann–Whitney test for panel (**i**).

To test whether the low numbers of bacteria phagocytosed (Fig. 2) was due to a lack of contact between *S. aureus* and macrophages, experimental plates were centrifuged after addition of bacteria to bring *S. aureus* into immediate contact with the phagocytes. After 2.5 h post-infection, there was an increase in the number of phagocytosed *S. aureus* bacteria (Fig. 3a,b). Without centrifugation (Fig. 2a,b), an average of 95.8% of macrophages had not phagocytosed any bacteria at an MOI 5, while 64% had not phagocytosed any at MOI 50. With centrifugation (Fig. 3a), the average percentage of macrophages which had not phagocytosed *S. aureus* was 72.3% for MOI 5, and 16.1% for MOI 50. Notably, centrifugation enhanced the numbers of macrophages which had phagocytosed MOI 5 *S. aureus* to similar levels to that of MOI 50 without centrifugation. This allowed investigation of increased phagocytosis levels without the possibility of overloading the system with a large inoculum.

### Increasing *S. aureus* inoculum does not increase bacterial mass numbers

As centrifugation enhanced the number of phagocytosed *S. aureus*, effectively increasing phagocytosed bacteria tenfold, the effect this had on mass formation was tested (Fig. 3a,b). Despite higher initial phagocytosis, a similar mass outcome was observed with centrifugation and otherwise identical infection conditions (Fig. 3c). The number of average single-colour masses per well remained at approximately 5, with dual-colour mass numbers remaining significantly lower. This supports clonal mass formation originating from some macrophages which have internalised a single bacterium.

To examine this further, the *S. aureus* inoculum was increased to a total MOI of 10, 20, and 50 (Fig. 3d,e,f), without centrifugation. Interestingly, the number of masses remained similar to that for MOI 5 *S. aureus* without centrifugation (Fig. 1b), with approximately 10 average total single-colour masses/well and < 1 average dual-colour mass/well (Fig. 3g,h). This suggests there is a limit to the number of masses which can form, which is not dependent on bacterial input numbers, but on the number of permissive host cells which can phagocytose bacteria but are unable to control them.

### Immune stimulation increases mass formation but not clonality

Macrophages can be polarised to give an M1 phenotype, resulting in a pro-inflammatory response^5,18^. Stimulation using LPS and IFN-γ led to an increased level of mass formation when challenged with an MOI 5 of 1:1 differentially labelled cells, but single clone masses still significantly predominated over duals (Figure S2a). We have also recently described the “augmentation” phenomenon, whereby co-phagocytosis of *S. aureus* with non-pathogenic bacteria leads to a reduction of *S. aureus* killing inside macrophages and an increase in mass formation^17^. Here we tested the effect of augmentation of *S. aureus* with heat killed *Micrococcus luteus* on macrophages and found increased mass formation but maintenance of clonality (Figure S2b).

### A mathematical model reveals clonal mass formation is likely to occur in a stochastic manner

The biological data are clearly suggestive of the explanation for clonality during infection is due to single *S. aureus* being phagocytosed at the outset, rather than multiple cells being engulfed that are then mostly killed. Both scenarios would lead to an individual that founds the clonal population that then forms a mass, escapes the macrophage and go on to cause a systemic infection. In order to set our biological data within a quantitative and predictive framework, we developed a stochastic individual-based model of the process leading to clonal mass formation. Here, our two mixed populations used in the macrophage interaction experiments are described as blue or red (Figure S3). An example time-course of the total red and blue intracellular densities across all macrophages from one simulation run (Figure S4a) shows an initial rapid increase due to phagocytosis which abruptly stops at 2.5 h when extracellular bacteria are removed. There then follows 10–12 h of exponential decline as killing far outweighs intracellular replication. However, after 15–20 h the killing rates of most macrophages have declined such that one or two bacteria persist in a small number of macrophages. They are then able to replicate and by 25 h we see, in this particular case, that numbers of the red bacteria have started to rise, suggesting masses may be forming.

In Figure S4b,c,d,e,f we show the mean (solid bars) and standard deviation (whiskers) of macrophages that contain either blue, red or both (dual) bacteria at 25 h, averaged across 100 simulation runs. In each case we see that the persistence of intracellular bacteria is a rare, stochastic event, with generally only 1 or 2 of the 2000 macrophages containing intracellular bacteria at 25 h. Indeed, in some simulation runs there were no intracellular bacteria at 25 h at all. In supplementary Figure S5 we perform sensitivity analyses, showing that the result of rare mass formation (generally around 0.1% of cells) is robust to most parameter changes. It is particularly noticeable that large changes to the extracellular growth rate and MOI make little difference to the key prediction of rare mass formation. We find only large decreases in initial intracellular killing or the killing saturation time lead to significantly larger numbers of persisting bacteria (though we never found more than 5% of cells containing bacteria at 25 h for the parameters tested). Broadly we see that the ratios of the initial extracellular densities are reflected in the proportions of runs where one or other bacteria type ‘wins’, and we see no ‘dual’ cases where macrophages contain bacteria of both types. These results demonstrate how clonal mass formation can arise as a rare, stochastic event where macrophage killing is exhausted and intracellular replication is present but low.

## Discussion

*S. aureus* continues to be a major threat to human health, especially due to the spread of antibiotic resistance^19–21^. Whilst the availability of a vaccine to prevent infection in vulnerable groups remains an important goal, all attempts so far have failed^22^. In order to understand the correlates of protection it is important to determine the correlates of disease and thus the fundamentals of infection dynamics. Recent research has highlighted that *S. aureus* can reside intracellularly, in particular within phagocytes^12,23,24^. The innate immune system is crucial in controlling infection by *S. aureus,* where macrophages play a crucial role^5^. In the initial phase of infection macrophages are typically extremely effective in the control of *S. aureus* but in rare events can actually be the progenitors of microabscesses that can then go onto seed other body sites^6^. This, in part, likely explains the need for large inocula in animal models to pass through this immune bottleneck^6^. The chance of macrophage failure can be increased by augmenting the *S. aureus* inoculum with non-pathogenic microflora, as would happen during natural infection^11^ and that this failure results from reduced exposure of the phagocytosed *S. aureus* cell to ROS. The failure of *S. aureus* control by macrophages results in bacterial, clonal population expansion, which here we have examined using mixtures of marked strains. Co-infecting with two fluorescent reporter strains demonstrated that the majority of masses originate from one bacterium, as most of the resultant bacterial masses were single-colour. Changing the ratio of GFP:mCherry bacteria favoured formation of masses in the dominant colour, with few dual-colour masses forming across all experiments. This supports previous work^6,8^, including a study which showed that by 3 days post-infection, mice injected with a mixture of *S. aureus* strains were predominantly colonised by a single strain^7^. Employing an individual-based model (Figure. S3) provided a broadly underpinning, quantitative framework for our observations of the stochastic nature of the development of population clonality.

**Figure 4:**
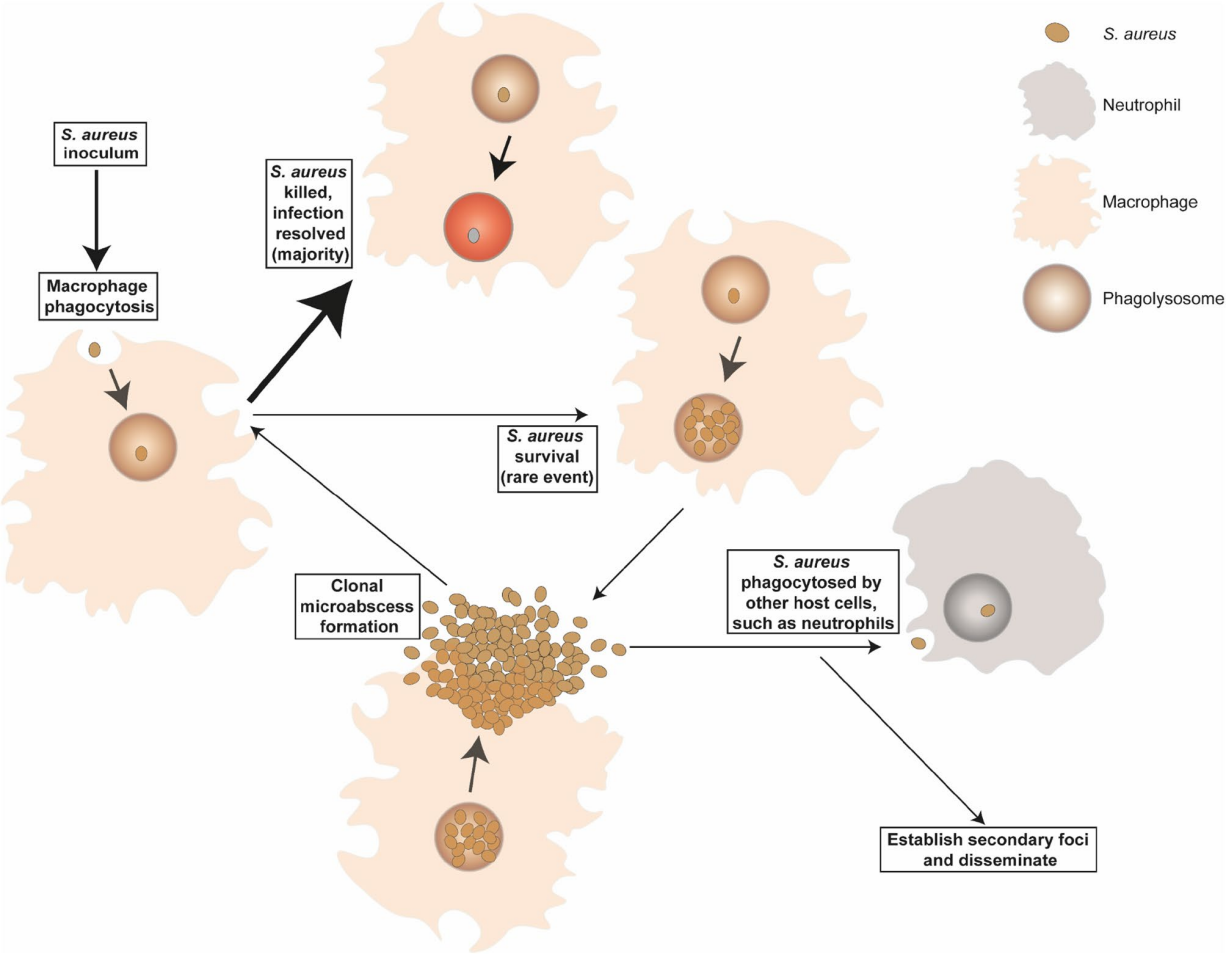
*S. aureus* clonal population dynamics. *S. aureus* enters the body and is phagocytosed by macrophages. The majority of *S. aureus* are killed in the phagolysosome vacuole, but a small proportion may survive to divide, expanding clonally until the bacteria escape from the macrophage to form clonal microabscesses, which can then either seed local abscesses, be re-phagocytosed by macrophages, or be phagocytosed by other immune cells, such as neutrophils. *S. aureus* may also establish secondary foci and disseminate to distant sites.

In probabilistic terms, if each macrophage were phagocytosing two bacteria, there would be a 25% chance of single-colour green or red masses and a 50% chance of dual-colour masses. If macrophages were phagocytosing three bacteria, there would be a 12.5% chance of either single colour green or red masses, and a 75% chance of dual-colour masses. As the number of bacteria phagocytosed by the macrophages increases, the chance of the phagocytosed bacteria all being of the same colour reduces, meaning the likelihood of clonal expansion and mass formation in a single colour is very low, further indicating that the majority of bacterial masses originate from macrophages which have phagocytosed one bacterium. This allows us to propose a model (Fig. 4), whereby systemic infection occurs via seeding from an individual bacterium. The key question was then, how does a single bacterium emerge from such a large inoculum? Investigations into the number of phagocytosed bacteria demonstrated that most macrophages which had phagocytosed *S. aureus* had only internalised 1 bacterium. This further lends support to the hypothesis that masses originate from clonal expansion of a single bacterium. Approximately 15% of liver cells are Kupffer cells^25,26^. There are an estimated 139 ± 25 million cells/g in the human liver^27^, with the average human adult male liver weighing ~ 1500 g^28^. As such, the average adult male liver has ~ 208 ± 37 × 10^9^ cells, and if 15% of these are Kupffer cells, then there are approximately 31 ± 6 × 10^9^ Kupffer cells. Although the levels of phagocytosis we demonstrate here may appear very low, the high numbers of Kupffer cells in the liver, coupled with the infectious dose for *S. aureus* being estimated at 10^5^ bacteria^29^, indicates that the actual MOI in human infection is likely very low. Added to this, Kupffer cells are not evenly distributed throughout the liver, mainly residing at sinusoids, the sites where blood, and therefore *S. aureus*, drains into the liver^30^. Macrophages are also important in skin and soft tissue infections where similar low numbers of infecting *S. aureus* are likely^31,32^. As we all are exposed to *S. aureus* regularly, as evidenced by circulating antibodies^33^, this alludes to the power of macrophages in preventing infection progression. However systemic infections can occur, particularly if there is a regular seeding of the blood via a biofilm on an indwelling device. The question then arises: why do a small number of macrophages fail to control *S. aureus*? Increasing the number of bacteria phagocytosed by macrophages as a result of a greater MOI or centrifugation to enhance contact, did not appear to lead to a greater number of masses. This may suggest only a subset of macrophages are permissive for *S. aureus* survival. It is possible that the RAW264.7 macrophages were unable to control *S. aureus* due to low levels of activation, however, it has been shown that stimulation of THP-1 macrophages with IFN-γ only led to a slight increase in intracellular killing, without ablating intracellular persistence of *S. aureus*^16^. Here we found no effect of macrophage stimulation using LPS and IFN-γ on clonality. Together these would suggest that intracellular survival of *S. aureus* and subsequent clonal expansion is not due to inadequate macrophage priming, also supported by work demonstrating similar inabilities of macrophages in vivo to control *S. aureus* within mice or zebrafish^6,8^. Macrophage heterogeneity could arise via several routes including differential levels of phagocyte maturity or altered NADPH activation kinetics. The addition of augmenting material increased the likelihood of mass formation but did not alter clonality. Augmentation occurs by protection of *S. aureus* in phagocytes from killing by ROS^17^, further supporting a role for phagocyte heterogeneity in the killing capacity.

The observations of clonal expansion from within a single macrophage (Fig. 4) has important implications for interventions such as vaccination and antibiotic therapy. The establishment of infection involves crucial intersections of bacterial and host biology. These are juxtaposed with stochastic processes that are dependent on ratios, and finite numbers, of host phagocytes and invading pathogens and coupled with other microflora as part of augmentation. It is only by unravelling this complex interplay that we can begin to define the correlates of disease, from the initial interaction to outcome in favour for the host or pathogen and in doing so determine the fundamental principles of disease.

## Methods

Where appropriate, all experimental protocols were approved by University of Sheffield committees.

### Bacterial strains and culture methodology

Microbial strains used in this study are listed in Table 1.

**Table 1:** Bacterial strains used in this study.

Species	Strain	Description	Culture conditions	Reference
*Staphylococcus aureus*	NewHG-mCherry (SJF 4442)	pMV158-mCherry *lysA::ery lysA* +	Tryptic Soy Broth (TSB), Erythromycin 5 µg/mL Tetracycline 5 µg/mL, at 37 °C	^6^
	NewHG-GFP (SJF 4440)	pMV158-GFP *lysA::kan lysA* +	TSB, Kanamycin 50 µg/mL, Tetracycline 5 µg/mL, at 37 °C	^6^
	NewHG-GFP (SJF4620)	*geh::Pma1M-GFP*	TSB, Kanamycin 50 µg/mL, at 37 °C	^6^

#### Bacterial growth curves

*S. aureus* GFP and mCherry strains (SJF4440 and 4442) were grown in broth culture overnight at 37 °C, with shaking (200 rpm). Cultures were adjusted to OD_600_ 0.05 in TSB with or without antibiotics, as indicated, and incubated at 37 °C, shaking (200 rpm). Samples were taken for serial dilution onto agar plates hourly until 7 h post-incubation, and then again at 24 h. Strains were incubated individually or in competitive culture, with colony forming units (CFUs) enumerated following incubation onto TSA agar plates with or without antibiotics, as indicated.

#### Cell culture

RAW264.7 cells (ATCC TIB-71), a macrophage cell line derived from a leukemic mouse were used. RAW264.7 cells were cultured in Dulbecco’s modified Eagle’s medium (DMEM, Sigma) supplemented with 2 mM L-glutamine (Sigma) and foetal bovine serum (Sigma) to a final concentration of 10% v/v. RAW264.7 cells were passaged upon achieving 70–80% confluence, with all experiments conducted between passages 5 and 20.

Monocyte derived macrophages (MDMs) were isolated as previously described^34^. Briefly, peripheral blood mononuclear cells were isolated by Ficoll Plaque (GE Healthcare) density centrifugation and seeded in 24 well plates at 2 × 10^6^ cells/well in RPMI-1640 media (Lonza) supplemented with 2 mM L-Glutamine, 10% v/v newborn calf serum (Gibco). Cells were incubated at 37 °C with 5% CO_2_. Non-adherent cells were removed after 24 h and adherent cells were fed fresh RPMI-1640 supplemented with 2 mM L-Glutamine and 10% v/v low endotoxin heat-inactivated foetal bovine serum (Biosera), which was replaced every 2–3 days. MDMs were used for experiments 14 days post-isolation.

Polarisation of RAW264.7 macrophages was performed by incubating cells with 20 ng/mL IFN-γ and 10 ng/mL LPS for 24 h. Macrophages were then incubated with fresh media for a further 24 h before use^35,36^.

#### Cell infection and *S. aureus* mass formation assay

The *S. aureus* mass formation assay was carried out as described previously^17^. Briefly, RAW264.7 cells were seeded into 96 well white micro-clear plates (Greiner) and incubated until reaching 80% confluence. RAW264.7 cells were infected with GFP and mCherry *S. aureus* (SJF4440 and 4442, respectively) at a 1:1 ratio and a total multiplicity of infection (MOI) of 5, unless otherwise stated. Bacteria were prepared as frozen stocks, as described previously^37^. Cells were incubated with bacteria for 2.5 h at 37 °C, 5% v/v CO_2_ before addition of media containing 20 µg/mL lysostaphin (Biosynexus). Cells were incubated for 1 h at 37 °C, 5% v/v CO_2_ before monolayers were washed three times with phosphate buffered saline (PBS, Fisher), replaced with fresh media and incubated to 25 h post-infection. Experimental wells were visualised using a 2 × objective lens and FITC and Cy3 filters on an ImageXpress Micro (Molecular Devices). MetaXpress high-content image acquisition and analysis software (Molecular Devices) was used to analyse average number of masses larger than 40 µm per well.

A variation of this experiment involved centrifugation of experimental plates at 100 g for 5 min at 37 °C immediately after addition of bacteria to macrophages, before incubation as described above.

#### Phagocytosis assays

In a modification of the mass formation assay, GFP *S. aureus* (SJF4620) were added to RAW264.7 cells at MOI 5 or 50 and incubated at 37 °C, 5% v/v CO_2_ for 0.5 h, 1 h, 1.5 h or 2.5 h. Media containing 20 µg/mL lysostaphin was added to wells and incubated for 0.5 h. Wells were washed three times with PBS before staining with CellMask™ Orange Plasma membrane Stain (Fisher) according to manufacturer’s instructions. Cells were fixed in 2% w/v paraformaldehyde (Sigma) for 1 h. Wells were washed three times in PBS, stained with 300 nM DAPI (Fisher), again washed in PBS and imaged on ImageXpress Micro, using a 20 × objective lens and DAPI, FITC and Cy3 filters. A custom module editor was created in MetaXpress high-content image acquisition and analysis software which identified the macrophages by fluorescence and counted the number of GFP bacteria within each macrophage.

In a variation of this experiment, experimental plates were centrifuged at 100 g for 5 min after addition of bacteria and incubated for 2.5 h prior to fixation and staining, as above. For “augmentation” experiments, heat killed *M. luteus* was prepared as previously described^17^, and added to RAW264.7 cells at an MOI of 50.

#### *S. aureus* macrophage infection timelapse

Timelapse experiments were performed as previously described^17^. Briefly, *S. aureus* NewHG-GFP was added to MDM cells in a 24 well plate on ice for 60 min, followed by incubation at 37 °C, 5% CO_2_ for 90 min. Media containing 20 μg/mL lysostaphin was added and incubated for a further 30 min. Cells were washed three times with PBS and fresh media was added. Cells were imaged every 10 min for 18 h on a Nikon Eclipse Ti microscope in a climate-controlled set-up (37CO_2_, Atmosphere: 5% CO_2_/95% air) with a × 20 Lambda Apo NA 0.75 phase contrast objective for brightfield or with a GFP filter. Images were captured with a Andor Neo-5.5-CL3 camera. Analysis was carried out using NIS elements (Nikon) and Fiji (ImageJ).

#### Mathematical model

We have implemented a stochastic individual-based model in Python. We assume two types of bacteria, blue and red, which are identical in all their rates (Supplementary Figure. 3). Extracellular bacteria (${B}_{ec}, {R}_{ec}$) replicate subject to standard logistic growth, with basic replication rate, ${r}_{ec}$, and carrying capacity, *K*. There are a fixed number of macrophages, ${M}_{0}$. Phagocytosis occurs as a mass action density-dependent process upon contact between macrophages and bacteria with coefficient, $\beta$. We assume a saturating rate of phagocytosis, as is common in models of bacteria-cell interactions^13,15^, such that phagocytosis initially increases with increasing numbers of bacteria but then levels off, with basic phagocytosis rate, $\beta$, and half-saturation constant, *c*. This saturation occurs due to physical limitations on the amount of bacteria cells can phagocytose in a given time period. After being phagocytosed, the bacteria become intracellular (${B}_{ic},{R}_{ic}$). We assume intracellular bacteria can replicate with no limit (given the short time-periods focussed on) at a lower rate, ${r}_{ic}$. Intracellular bacteria can be killed by the macrophages at a rate, $\mu (t)$, which depends on the time since that particular macrophage was first infected. We assume the killing rate declines once a macrophage has phagocytosed its first bacterium, following previous work suggesting declining killing rates^15^, with a step-like function, such that killing is initially high for a short period and then rapidly declines towards 0. The deterministic equivalent of our model would be given by the following ordinary differential equations,

$$\begin{aligned} \frac{{dB_{ec} }}{dt} = & r_{ec} B_{ec} \left( {1 - \frac{{\left( {B_{ec} + R_{ec} } \right)}}{K}} \right) - \frac{{\beta M_{0} B_{ec} }}{{B_{ec} + R_{ec} + c}} \\ \frac{{dR_{ec} }}{dt} = & r_{ec} R_{ec} \left( {1 - \frac{{\left( {B_{ec} + R_{ec} } \right)}}{K}} \right) - \frac{{\beta M_{0} R_{ec} }}{{B_{ec} + R_{ec} + c}} \\ \frac{{dB_{ic} }}{dt} = & r_{ic} B_{ic} + \frac{{\beta M_{0} B_{ec} }}{{B_{ec} + R_{ec} + c}} - \mu \left( {t - \tau } \right)B_{ic} \\ \frac{{dR_{ic} }}{dt} = & r_{ic} R_{ic} + \frac{{\beta M_{0} R_{ec} }}{{B_{ec} + R_{ec} + c}} - \mu \left( {t - \tau } \right)R_{ic} \\ \end{aligned}$$

We use a commonly used ‘Hill-function’ form for the saturating killing. Denoting the initial killing rate as ${\mu }_{0},$ the time at which a macrophage was first infected by $\tau$, and the time point at which the killing rate is halved, $\gamma$ , the form of the killing function, $\mu$, in each macrophage is given by,

$$\mu \left( {t - \tau } \right) = \mu_{0} \left( {1 - \frac{{\left[ {t - \tau } \right]^{2} }}{{\left[ {t - \tau } \right]^{2} + \gamma^{2} }}} \right)$$

The deterministic approach would assume large population sizes. As our focus is on the outcome when bacterial populations are low, we instead implement a stochastic version of the model, using a direct-method (Gillespie) algorithm^38^ to translate the ordinary differential equations into a discrete, probabilistic model. It is worth noting that when phagocytosis occurs in the model, a macrophage is initially selected to phagocytose, with then an extracellular red or blue bacterium selected to become internalised according to their relative densities.

### Statistical analysis

Statistical analysis was carried out in Prism 9.2.0 (GraphPad), with *p* < 0.05 considered significant. Experiments were analysed using Kruskal–Wallis one-way analysis of variance (ANOVA) tests with Dunn’s post-test or a two-tailed Mann–Whitney test, as indicated.

### Ethics statement

MDMs were derived, with informed consent, from the blood of healthy volunteers in accordance with guidelines from the South Sheffield Research Ethics Committee (07/Q2305/7).

## Data and code availability

All data from this study is available within the paper or supplementary material. All figure data are deposited on the ORDA database (http://dx.doi.org/10.15131/shef.data.18551081). Python code available on Github (https://github.com/abestshef/Clonality).

## Supplementary Information

Supplementary Video 1.

Supplementary Information 1.
