# Supplementary material for: Clonal population expansion of Staphylococcus aureus occurs due to escape from a finite number of intraphagocyte niches
Source: Sci Rep. 2023 Jan 21;13:1188. doi: 10.1038/s41598-023-27928-2 (PMC9867732; doi:10.1038/s41598-023-27928-2)
Supplement: Supplementary file 2 — Supplementary Information 1. [file 41598_2023_27928_MOESM2_ESM.pdf]

Movie S1 The expansion of an *S. aureus* mass within a macrophage.

Video showing the expansion of an *S. aureus* NewHG-GFP mass (left panel, green) within a monocyte derived macrophage (centre panel, grey, brightfield) with overlay of NewHG-GFP and MDM (right panel). The mass grows resulting in the lysis of the macrophage. Video represents a period of 14 hours. Scale bars represent 20 µm.

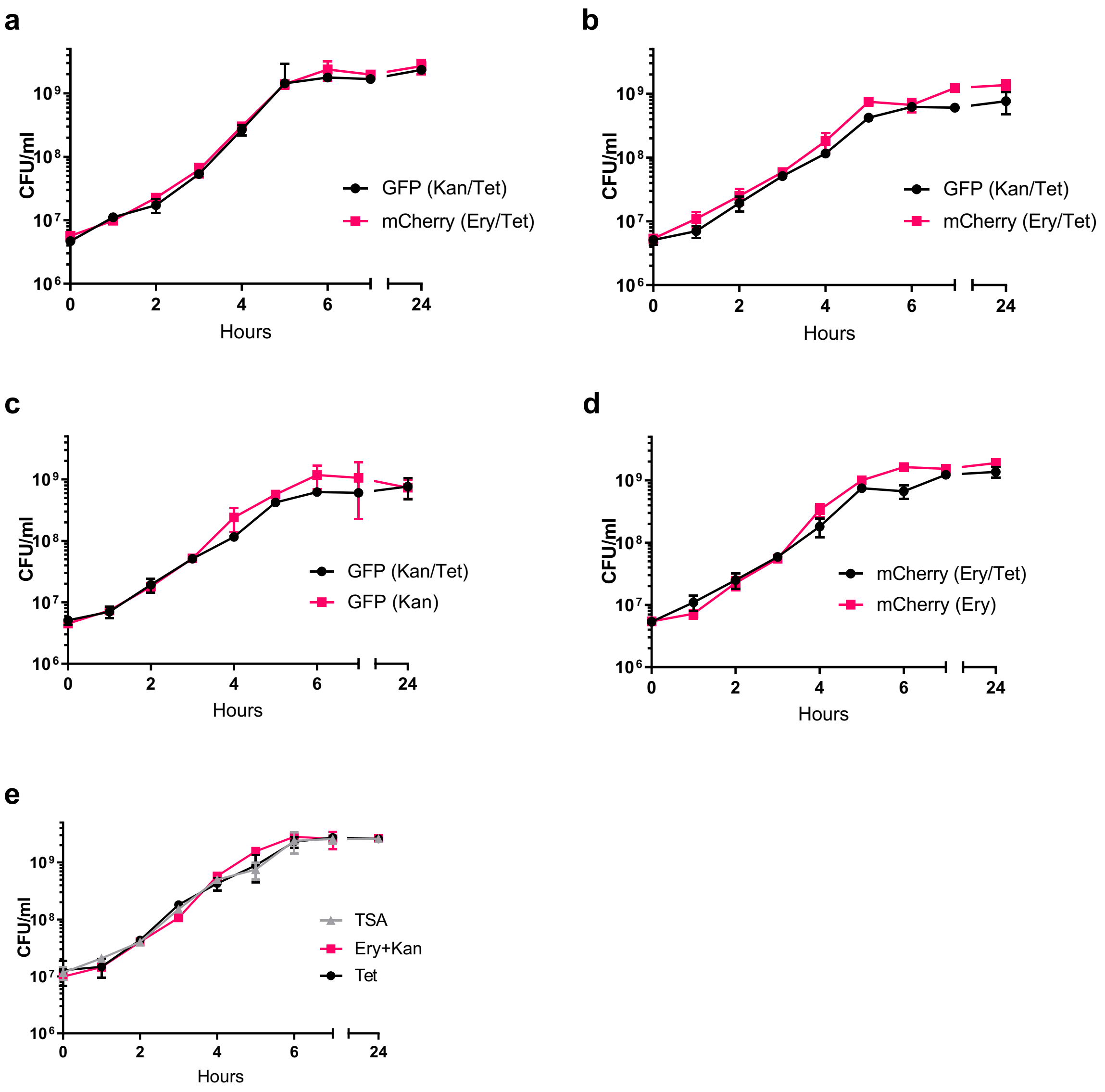

Supplementary Figure 1 Bacterial growth is comparable between GFP and mCherry marked strains.

a, Growth curves of GFP and mCherry fluorescent reporter strains grown in individual cultures; **b,** Competitive growth curves of GFP and mCherry fluorescent reporter strains grown together in a mixed culture; **c,** Growth curves of the GFP fluorescent reporter strain grown in individual culture, CFUs enumerated on agar plates in the presence of Kanamycin (GFP Kan) or Kanamycin and Tetracycline (GFP Kan/Tet); **d,** Growth curves of the mCherry fluorescent reporter strain grown in individual culture, CFUs enumerated on agar plates in the presence of Erythromycin (GFP Ery), or Erythromycin and Tetracycline (GFP Ery/Tet); **e,** Growth curves of the GFP and mCherry fluorescent reporter strains grown in individual culture, CFUs enumerated on agar plates in the presence of Erythromycin or Kanamycin

with counts added together, (Ery+Kan), Tetracycline (Tet), or no antibiotics. Each growth curve represents three biological repeats, showing mean ± standard deviation.

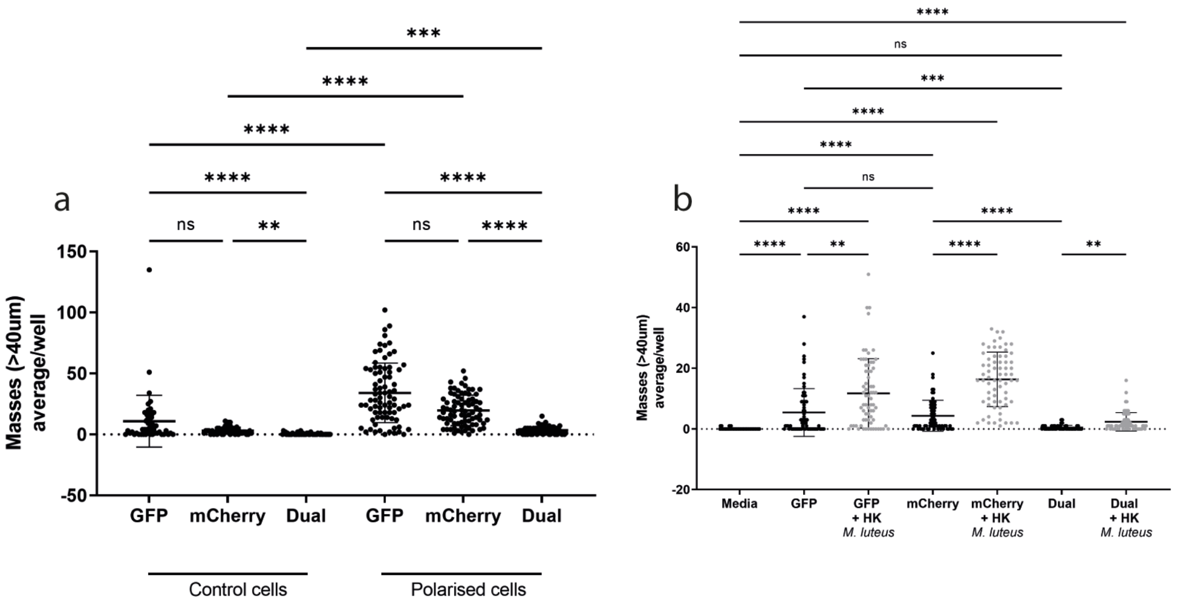

Supplementary Figure 2 Effect of interventions on clonal mass formation.

a-b, GFP and mCherry *S. aureus* were added to RAW264.7 cells in a 1:1 ratio (total MOI=5), spun and then incubated until 25 h post-infection. The number of single- or dual-colour bacterial masses detected at 25 h post-infection is indicated, showing mean values ± standard deviation, $^{**}p<0.005$, $^{****}p<0.0001$, as determined using a Kruskal-Wallis test with Dunn's post-hoc test. **a,** RAW264.7 macrophages were incubated with 20 ng/mL IFN-γ and 10 ng/mL LPS for 24 h ('polarised cells') or blank media ('control cells'), then incubated with fresh media for 24 h before infection with GFP and mCherry *S. aureus* (n=2). **b,** RAW264.7 macrophages were infected with GFP and mCherry *S. aureus* in the presence (grey circles) or absence (black circles) of heat killed *M. luteus* (n=4).

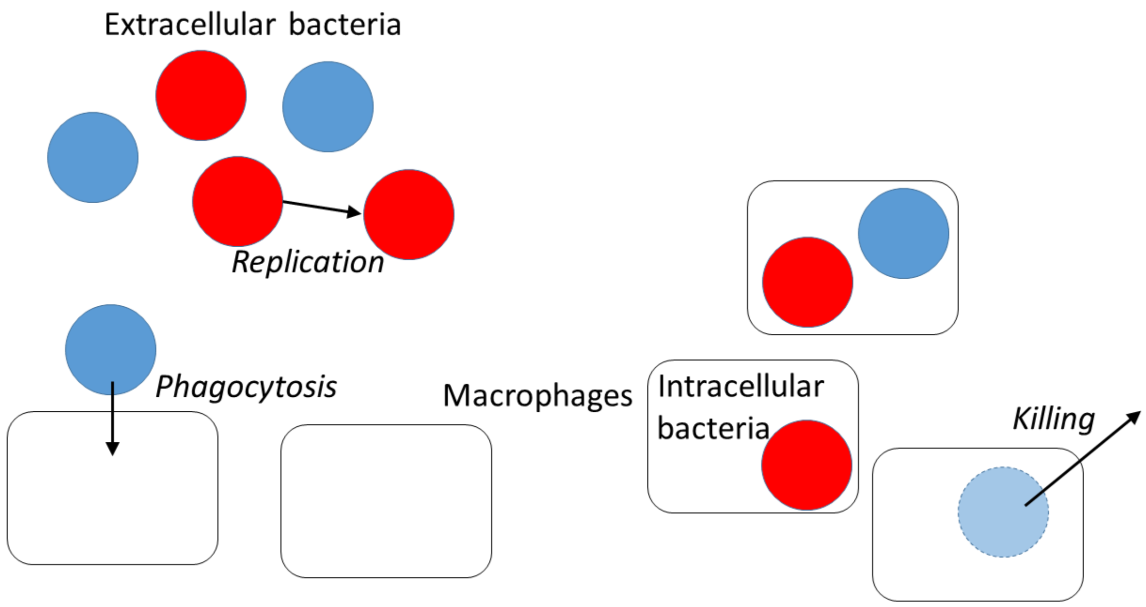

Supplementary Figure 3 Schematic of the mathematical model.
Coloured circles represent the two types of bacteria (red and blue) and open boxes the macrophages. Arrows indicate mechanisms included in the model. Extracellular bacteria of each type can replicate (top-left) and be phagocytosed by macrophages (bottom-left). Once phagocytosed, bacteria become intracellular, where they remain until killed (bottom-right).

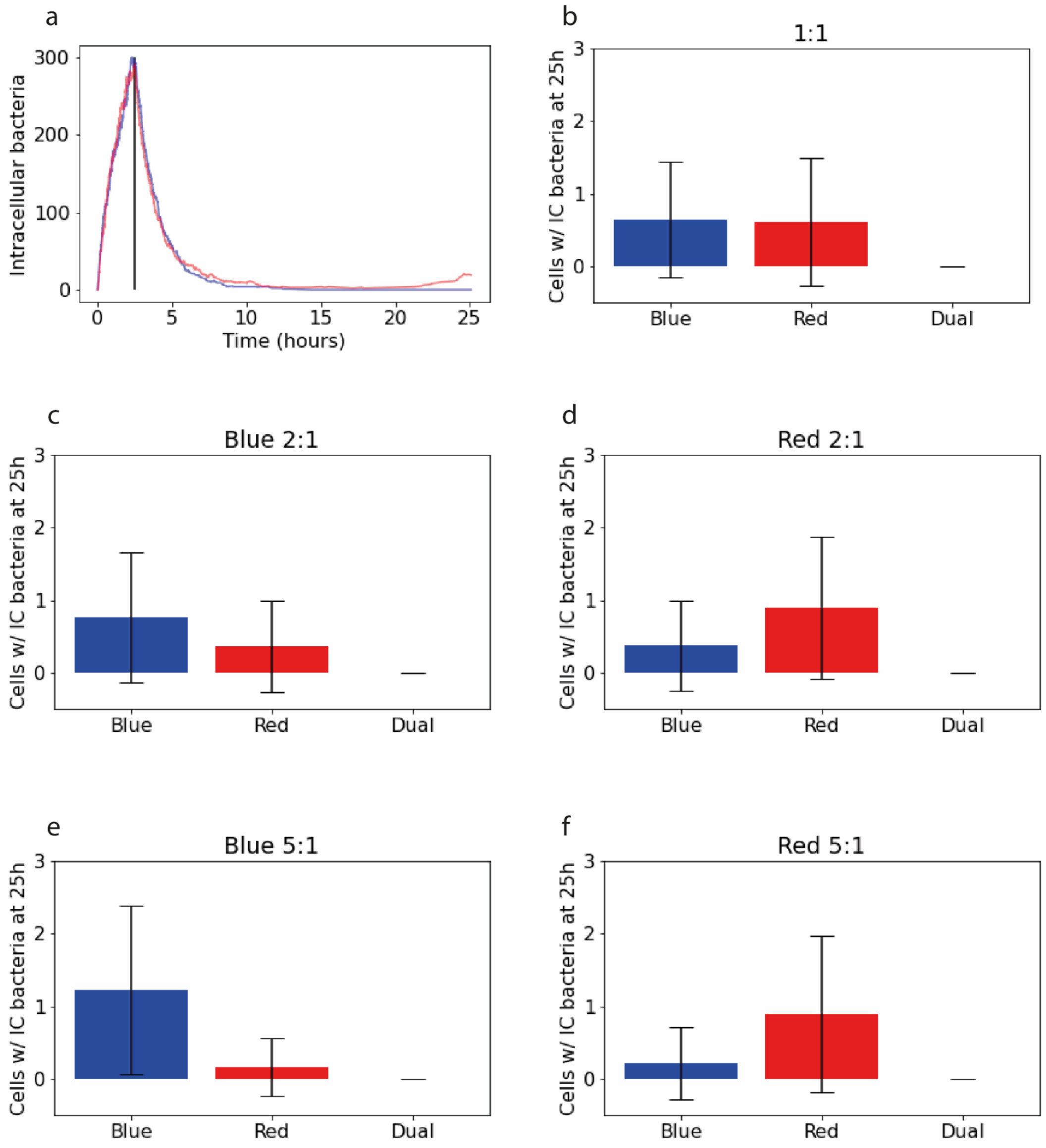

Supplementary Figure 4 Mathematical modelling of clonal mass numbers.

a, Time course of total intracellular bacteria from one simulation run of the stochastic model. The vertical bar at 2.5h marks where extracellular bacteria were removed (1:1 ratio of blue and red bacterial populations. **b-f,** Output from the stochastic model, showing the mean (solid bars) and standard deviation (whiskers) of the number of macrophages containing blue, red, or both (dual) bacteria at 25 hours, averaged across 100 simulation runs. Initial ratios of extracellular bacteria are used in each plot: **b** (1:1), **c** (2:1 blue), **d** (2:1 red), **e** (5:1 blue), **f** (5:1 red). Parameter values used: $r_{ec} = 0.5, r_{ic} = 0.25, K = 5 \times 10^4, \beta = 0.25, c = 100, \mu_0 = 1, \gamma = 9$ and initially 10^4 total extracellular bacteria and 2×10^3 macrophages.

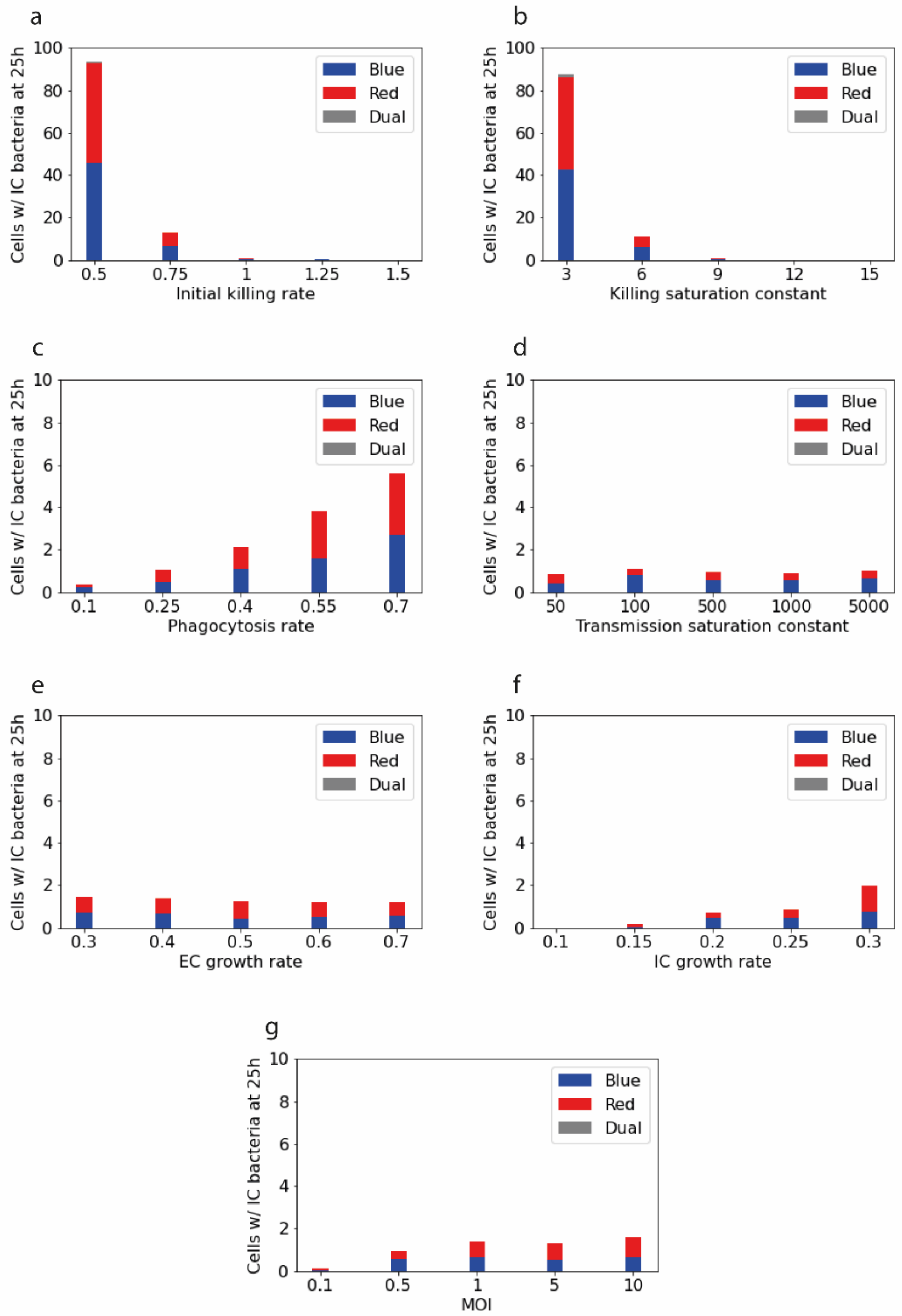

Supplementary figure 5 Sensitivity analysis of model.

Model results showing the number of macrophage cells with blue, red or both (dual) bacteria present at 25 hours as parameters are varied. Default parameter values in all plots are as in Figure 5 in the main text and an initial 1:1 ratio of blue:red bacteria is assumed in all cases.
